# A comparsion study between debridement, antibiotics, and implant retention and two-stage revision total knee arthroplasty for the management of periprosthetic joint infection occurring within 12 weeks from index total knee arthroplasty

**DOI:** 10.1186/s13018-022-03218-x

**Published:** 2022-06-27

**Authors:** Yanchao Zhang, Zhisen Gao, Ti Zhang, Yu Dong, Zhuoqi Sheng, Fei Zhang, Yonggang Zhou, Lingfei Guo

**Affiliations:** 1 Medical School of Chinese PLA, Beijing, 100853 China; 2 Department of Orthopedics, The First Medical Center, Chinese People’s Liberation Army General Hospital, Fuxing Road, Haidian District, Beijing, 100048 China; 3 Senior Department of Orthopedics, The Fourth Medical Center of PLA General Hospital, Beijing, 100048 China; 4 Medical School of Nankai University, Tianjin, 300071 China; 5 Department of Orthopedics, the Eighth Medical Centre, Chinese People’s Liberation Army General Hospital, Heishanhu Road, Haidian District, Beijing, 100091 China

**Keywords:** Total knee arthroplasty, Periprosthetic joint infection, Revision

## Abstract

**Background:**

Managing periprosthetic joint infections are variable in practices. Debridement, antibiotics, and implant retention (DAIR) is one of the favorable interventions. Given that the success rate of the two-stage revision total knee arthroplasty (rTKA) might be overestimated. The purpose of this study is to compare the success rate between DAIR and standard two-stage rTKA with a comparable intervention time.

**Methods:**

We retrospectively reviewed the consecutive knee periprosthetic joint infection cases which underwent DAIR or two-stage rTKA (all procedures were performed by the senior author) within 12 weeks since their primary TKA between July 2009 and October 2019. Average follow-up was 72.20 ± 40.70 months (range 29–148 months) in the DAIR group compared to 89.14 ± 43.06 months after spacer insertion (range 29–163 months) in the two-stage revision group (*P* = 0.156). According to different interventions, demographic data; timing of surgical intervention; hospital for special surgery knee score; and success rate were collected and compared between the DAIR group and two-stage revision group. Failure of treatment was based on the Delphi consensus and the fate of spacers. The pathogen types and failure cases were also recorded and analyzed.

**Results:**

Average follow-up was 72.20 ± 40.70 months (range 29–148 months) in the DAIR group compared to 89.14 ± 43.06 months after spacer insertion (range 29–163 months) in the two-stage revision group. Time from index surgery was 3.90 ± 2.92 weeks (range 0–12 weeks) in the DAIR group, and 5.11 ± 2.86 weeks (range 0–12 weeks) in the 2-stage exchange group, respectively. The success rate was 70.0% and 75.0% in the DAIR group and two-stage revision group, respectively. But no significant differences were observed between the two groups.

**Conclusion:**

DAIR demonstrated comparable effectiveness with two-stage rTKA. We recommended DAIR as a choice for patients with current infection within 12 weeks after primary TKA. For methicillin-resistant staphylococcal infections and fungal infections, two-stage rTKA might be preferred.

## Background

Around the world, preventing and managing periprosthetic joint infections (PJIs) are variable in practices. Surgical treatments include debridement, antibiotics, and implant retention (DAIR); single- or two-stage revision; arthrodesis; and amputation [1]. Many surgeons termed two-stage revision total knee arthroplasty (rTKA) as the “gold standard” treatment [2, 3]. But recent studies have doubted the success rates of this strategy, because some patients may need an interim spacer exchange or even never undergo the second stage [4, 5].

By comparison, benefits of DAIR include retaining implants, preserving bone stock, shorter procedure duration, reducing intraoperative fractures, and rehabilitating faster [6, 7]. Barry et al. [8] found DAIR is as effective as two-stage rTKA with a success rate of 62.5%. However, in this comparative study, symptom duration showed a significant difference between the DAIR group and two-stage revision group. Symptom duration is an independent risk factor associated with failure of knee PJI treatment [9]. It might indicate that the different symptom durations resulted in this outcome, rather than the interventions themselves. Both symptom duration and initial arthroplasty duration reflect intervention time. To control the variable of intervention time, as the International Consensus Meeting on Periprosthetic Joint Infection (ICMPJI) recommended [10], we reviewed the PJIs within 12 weeks of index primary arthroplasty. This study designed to compare the success rate between DAIR and standard two-stage rTKA in patients within 12 weeks of prosthesis implantation.

## Materials and methods

After the institutional review board’s approval, a retrospective review was performed on the consecutive cases which underwent DAIR or two-stage rTKA within 12 weeks since their primary TKA between July 2009 and October 2019. All procedures were performed by the senior author. PJI was diagnosed according to the criteria of the Musculoskeletal Infection Society [11]. The exclusion criteria were (1) PJI happened beyond the primary TKA. (2) Patients with clinical or radiographic evidence of implant loosening. (3) Patients who had prior septic arthritis, bone neoplasms. (4) Patients who died between primary TKA and interventions. (5) The first stage rTKA process outside of our hospital. (6) Incomplete patient information. Based on these criteria, 54 patients (56 knees) were included in the final cohort. Preoperative diagnosis included osteoarthritis (49 knees), traumatic osteoarthritis (2 knees), rheumatoid arthritis (3 knees), and pigmented villonodular synovitis (2 knees). Among the cohort, 20 patients (20 knees) were in the DAIR group and 34 patients (36 knees) were in the two-stage revision group. Charlson comorbidity index [12]—including cardiovascular and cerebrovascular disease, pulmonary disease, digestive system disease, rheumatological disease, diabetes, malignancy, and AIDS—was used to reduce potential confounding. Patient demographics are shown in Table 1.

**Table 1:** Patient demographics

	DAIR	Two-stage	*P* value
Age (years)	65.10 ± 6.63	67.68 ± 7.52	0.210
Gender			0.983
Male	7	12	
Female	13	22	
BMI (kg/m^2^)	26.77 ± 3.86	26.67 ± 3.05	0.912
Charlson index^a^	0.75 ± 0.72	0.71 ± 0.76	0.834

DAIR, Debridement, antibiotics, and implant retention group; Two-stage, Two-stage revision group; BMI, body mass index

^a^Charlson comorbidity index

Infecting organisms were recorded and analyzed according to the results of preoperative aspiration and intraoperative pathology. Culture negative was defined as being culture-negative result for all samples (at least three consecutive negative culture results) [13]. And all DAIR procedures included polyethylene exchange.

Postoperatively, all patients received antibiotic therapy based on the culture results and antibiotic susceptibility. For DAIR group, patients were firstly treated with 6 weeks of intravenous antibiotics followed by 12-week oral antibiotics. For two-stage revision group, antibiotic therapy was divided into two parts. After placing an articulating antibiotic impregnated cement spacer, 6-week intravenous antibiotics and 6-week oral antibiotics were applied. After the first step of antibiotic therapy, patients had a 2 to 3 weeks’ antibiotic holiday. The interval was established by patient symptoms and laboratory examinations after the antibiotic holiday. The laboratory examinations include routine joint fluid test, erythrocyte sedimentation rate (ESR), C-reactive protein (CRP), interleukin-6 (IL-6). Post reimplantation (the second stage of two-stage rTKA), the antibiotic regimen included 4-week intravenous antibiotics and 8-week oral antibiotic treatment. Failure of treatment was based on the consensus definition for success after PJI treatment [14] and the fate of spacers: (1) failed infection eradication, characterized by the presence of a sinus tract, drainage, pain, or infection recurrence caused by the same organism strain; (2) subsequent surgery for infection after the intervention; (3) failure that occurs between the two stages of two-stage rTKA; (4) medically unfit for reimplantation; (5) PJI-related mortality. Postoperative clinical and radiological data were obtained at 3 months, 6 months and annually thereafter. Hospital for special surgery knee score (HSS), complications, and recurrence of infection were collected at each follow-up. Complete data were available for all patients.

### Statistics

The mean values and ranges were calculated for demographic data and presented using mean ± standard deviation with ranges. Categorical variables were described with percentages. T test was used to compare the difference between the two interventions. Chi-square analysis and Fisher exact test were used for gender compositions and success rates. Statistical significance was defined as *P* < 0.05. All statistical analyses were conducted with SPSS version 24.0 (IBM Inc., Armonk, New York).

## Results

The basic level (including age, gender composition, BMI, and Charlson comorbidity index) showed no difference between the DAIR group and two-stage revision group. Average follow-up was 72.20 ± 40.70 months (range 29–148 months) in the DAIR group compared to 89.14 ± 43.06 months after spacer insertion (range 29–163 months) in the two-stage revision group (*P* = 0.156). Time from index surgery was 3.90 ± 2.92 weeks (range 0–12 weeks) in the DAIR group, and 5.11 ± 2.86 weeks (range 0–12 weeks) in the two-stage exchange group, respectively. But no significant differences were observed between the two groups (*P* = 0.137) (Table 2).

**Table 2:** Data of the latest follow-up

	DAIR	Two-stage	*P* value
Follow-up (months)	72.20 ± 40.70	89.14 ± 43.06	0.156
Time from index Surgery (weeks)	3.90 ± 2.92	5.11 ± 2.86	0.137
Preop HSS	51.50 ± 13.45	46.67 ± 16.64	0.271
Postop HSS	65.50 ± 13.66	65.39 ± 15.46	0.979
Success rate	70.0%	75.0%	0.686

DAIR, Debridement, antibiotics, and implant retention group; Two-stage, two-stage revision group; preop, Preoperative; postop, postoperative; HHS, hospital for special surgery knee score

Table 3 lists infecting organisms according to the results of preoperative aspiration and intraoperative pathology. The most common monomicrobial infecting organisms in the DAIR group were coagulase-positive staphylococcus (25.0%) and Streptococcus (15.0%). Multiple bacteria infected three patients (15.0%). The culture results were negative in five patients (25.0%). There were one case of methicillin-resistant staphylococcus epidermidis (MRSE 50%) and one case of methicillin-resistant staphylococcus aureus (MRSA 20%). Comparing with two-stage revision group, coagulase-negative staphylococcus (19.4%), fungus (13.9%) and coagulase-positive staphylococcus (8.3%) are the most common infecting organisms. Multiple bacteria infected three patients (8.3%). The culture results were negative in 13 patients (36.1%). There were three cases of MRSE (43%) and one case of MRSA (33%). One case of MRSA in DAIR group and one case of MRSE in two-stage revision group failed.

**Table 3:** Microorganism spectrum

Type of pathogen	Number (%)	DAIR		Two-stage	
		Success	Failure	Success	Failure
Coagulase-negative Staphylococcus	9 (16.1)	2	0	4	3
Coagulase-positive Staphylococcus	8 (14.3)	3	2	2	1
Mycobacterium Tuberculosis	3 (5.4)	0	2	1	0
*Escherichia coli*	2 (3.6)	0	0	1	1
Streptococcus	3 (5.4)	3	0	0	0
Enterococcus	1 (1.8)	0	0	1	0
Pseudomonas Aeruginosa	1 (1.8)	0	0	1	0
Fungus	5 (8.9)	0	0	4	1
Polymicrobial Infection	6 (10.7)	2	1	2	1
Culture negative	18 (32.1)	4	1	11	2
Total	56 (100)	14	6	27	9

DAIR, Debridement, antibiotics, and implant retention group; Two-stage, Two-stage revision group

The HSS scores of both groups improved in the latest follow-up, indicating satisfactory clinical efficacy. But we found no difference in preoperative HSS score and postoperative HSS score between the two groups.

The success rate was 70.0% and 75.0% in the DAIR group and two-stage revision group, respectively. Six patients (6 knees) reinfected in the DAIR group, including coagulase-positive staphylococcus (*n* = 2), mycobacterium tuberculosis (*n* = 2), polymicrobial infection (*n* = 1), and culture-negative PJI (*n* = 1). Among the six recurrences of PJI, three patients underwent a second DAIR, two of them chose two-stage rTKA, and one patient with a recurrent sinus tract hadn’t sought any treatment when we did the telephone follow-up.

In the 34 patients (36 knees) who underwent an intended two-stage revision rTKA, only 28 (30 knees) completed the second stage. Of the six patients who never undergo the second stage following the spacer placement, four patients medically unfit reimplantation and retained spacers, two patients retained spacers with acceptable function. After stopping antibiotics, those two patients didn’t have the infectious symptom recurrence and the laboratory examinations were normal. Their treatment was considered to be successful. Treatment success in this subset of patients was 75.0%. Among the nine failed cases, four patients never underwent reimplantation with persistent infections, four had another reinfection and one patient died due to PJI. Three knees of the failed cases were infected with coagulase-negative staphylococcus, one knee with coagulase-positive staphylococcus, one knee with escherichia coli, one knee with fungus, one knee with polymicrobial infection, and two knees with negative culture.

## Discussion

We found that DAIR had a similar success rate and functional outcome with the patients who underwent two-stage revision. Easier surgery, preservation of bone stock, and lower morbidity make DAIR a better choice for some patients. Reports on success rates of DAIR are inconsistent, ranging from 0 to 90% [15–18]. Organism type, host factors, timing of intervention, and antibiotic treatment might contribute to the reported differences. In our study, the DAIR group had a success rate of 70%. We found no significant difference in success rates between the DAIR group and two-stage revision group.

For two-stage rTKA, many studies reported that the success rate was more than 80% [19–22]. But scholars have put forward evidence to question this view recently. Tan T.L et al. found that nearly 17% of patients may need an interim spacer exchange lacking infection control [23]. Another study demonstrated that 18% of PJIs may never complete reimplantation (the second stage of two-stage rTKA) [24]. Ford [5] taking the patients who never underwent reimplantation into account, the success rate decreased from 72.7 to 60%. In our two-stage revision group, we termed the fate of spacers as the outcome indicator rather than infection clearance, which likely overestimate the success of this treatment.

Timing of surgical intervention is one of the influence factors affecting success rate of PJI treatment [25–28]. But the optimal timing of performing DAIR is under debate. Mirza et al. [29] recommended 2 weeks as the time window, describing that the biofilm is not mature and bacteria are more susceptible to microbiological agents within 2 weeks of infection. However, an animal study verified that biofilm formation was evident in all specimens from animals within hours [30]. In our study, more than 91% of patients had symptoms for more than 2 weeks. It indicated that most patients had formed biofilm before they started interventions. And we should extend the intervention time inclusion criteria of DAIR moderately, thus, more patients could receive less invasive surgery without implant removal. Ottesen et al. [31] displayed a 90% success rate in the patients revised within 12 weeks compared 60% in the patients revised over 12 weeks. It showed that 12 weeks might be the accepted intervention time cut-point.

Type of pathogen, higher microorganism virulence, biofilm formation, and resistance to antibiotics might contribute to failed treatments. Aboltins [32] concluded that the success rate of treating staphylococcal prosthetic joint infections with DAIR was 80%. However, in patients with methicillin-resistant staphylococcal infections, the total success rate of DAIR was 18% [33]. In our study, one case of MRSA in DAIR group failed (50%). However, small sample size might lead to statistical bias. Given the low success rate of DAIR in this situation, Bradbury T et al. recommended two-stage rTKA instead. Small number of subjects and elderly patients might limit its reliability and external validity. In a study of Marculescu et al. [34], the 2-year survival rate of polymicrobial was lower but not statistically significant different than of monomicrobial PJI treated with DAIR (52.7% vs 54.0%). It indicated that multiple bacterial infections suit the treatment of DAIR. Jacobs et al. [35] found culture-negative DAIRs were not related to any complications during follow-up, overtreatment of a suspected PJI seems to do no significant harm with respect to implant failure. And all culture-negative cases were treated successfully. In our study, the success rate of culture-negative cases with DAIR is 80%, which is similar to the success rate of two-stage revision TKA (84%). Findings of a large-scale multicenter study on prosthetic joint infections caused by fungal pathogens supported the notion that two-stage rTKA may benefit for the majority of patients [36]. DAIR had a limited role and should be reserved for the healthy host with excellent soft tissues and a truly acute infection. Our previous study was consistent with this idea [37]. Only our two-stage revision group included fungal PJIs and it showed a good outcome (with a success rate of 80%). We would recommend two-stage rTKA to treat fungal PJIs. But further studies should be conducted to confirm this hypothesis.

There has been a debate regarding the outcomes of further treatments when DAIR had been carried out previously. In the study of Rajgopal et al. [38], the survival rates were lower for patients in the previous DAIR group (79.5% at 2 years and 76.13% at 10 years) compared to the two-stage revision group (85.4% at 2 years and 84.4% at 10 years). It concluded that a failed prior DAIR results in higher failure rates. But it falsely enlarged the success rate of two-stage rTKA by only including the patients who had completed the second stage of reimplantation in their final study cohort. In addition, for many studies, the 10-year survival rate of 76.13% is a satisfactory result, which cannot prove that DAIR is not a good method. Kim et al. [39] found that, at mean follow-up of 6.2 years, the success rate was 72% in the failed DAIR group and 81% in the two-staged revision group. No significant difference was observed on survival analysis in both treatment groups. It means that failed DAIR does not compromise the success of further interventions in infected TKA.

Our study has certain limitations. First, this is a retrospective design, only the record information is available for the study. Second, a relatively small sample size limits statistical comparisons between different groups. Further multicenter prospective clinical trials and larger sample size are necessary to validate the outcomes. Third, the selection of antibiotics was based on culture result and antibiotic susceptibility. However, different organisms and antibiotics may produce variation in our treatment. Fourth, the senior author in our team, following a consistent treatment protocol, performed all enrolled cases (including DAIRs and two-staged rTKAs). Limited cases failed to form a matched cohort study between groups. However, we demonstrated that the basic level has no difference between our DAIR group and the two-stage revision group.

## Conclusion

In our study, the success rate of DAIR was 70%. DAIR demonstrated comparable effectiveness with two-stage rTKA. Additionally, advantages of DAIR include less trauma, relatively simple operation, preservation of bone reserve, quick recovery, and low postoperative morbidity. We recommended DAIR as a choice for patients with current infection within 12 weeks after primary TKA. According to our research it is acceptable to apply DAIR procedure in culture negative PJIs and in polymicrobial cases, but further investigations needed. For methicillin-resistant staphylococcal infections and fungal infections, two-stage rTKA may be preferred.

## Data Availability

All data generated or analyzed during this study are included in this published article.

## Abbreviations

DAIR: Debridement, antibiotics, and implant retention
TKA: Total knee arthroplasty
PJI: Periprosthetic joint infection
HSS: Hospital for special surgery knee score
